# Personalized breast cancer screening with selective addition of digital breast tomosynthesis through artificial intelligence

**DOI:** 10.1117/1.JMI.10.S2.S22408

**Published:** 2023-06-01

**Authors:** Victor Dahlblom, Anders Tingberg, Sophia Zackrisson, Magnus Dustler

**Affiliations:** a Lund University, Department of Translational Medicine, Diagnostic Radiology, Malmö, Sweden; b Skåne University Hospital, Department of Medical Imaging and Physiology, Malmö, Sweden; c Lund University, Department of Translational Medicine, Medical Radiation Physics, Malmö, Sweden; d Skåne University Hospital, Radiation Physics, Malmö, Sweden

**Keywords:** breast cancer screening, digital breast tomosynthesis, artificial intelligence, personalized screening

## Abstract

**Purpose:**

Breast cancer screening is predominantly performed using digital mammography (DM), but digital breast tomosynthesis (DBT) has higher sensitivity. DBT demands more resources than DM, and it might be more feasible to reserve DBT for women with a clear benefit from the technique. We explore if artificial intelligence (AI) can select women who would benefit from DBT imaging.

**Approach:**

We used data from Malmö Breast Tomosynthesis Screening Trial, where all women prospectively were examined with separately double read DM and DBT. We retrospectively analyzed DM examinations (n=14768) with a breast cancer detection system and used the provided risk score (1 to 10) for risk stratification. We tested how different score thresholds for adding DBT to an initial DM affects the number of detected cancers, additional DBT examinations needed, detection rate, and false positives.

**Results:**

If using a threshold of 9.0, 25 (26%) more cancers would be detected compared to using DM alone. Of the 41 cancers only detected on DBT, 61% would be detected, with only 1797 (12%) of the women examined with both DM and DBT. The detection rate for the added DBT would be 14/1000 women, whereas the false-positive recalls would be increased with 58 (21%).

**Conclusion:**

Using DBT only for selected high gain cases could be an alternative to complete DBT screening. AI can analyze initial DM images to identify high gain cases where DBT can be added during the same visit. There might be logistical challenges, and further studies in a prospective setting are necessary.

## 1 Introduction

Breast cancer screening is implemented in many countries to detect breast cancer at an earlier stage with better treatment possibilities and is usually recommended to be performed with two-view digital mammography (DM) with mediolateral oblique (MLO) and craniocaudal (CC) views of each breast.^1^^,^^2^ Digital breast tomosynthesis (DBT) has been shown to be superior to DM in cancer detection,^3^ but DBT is not yet widely accepted as a substitute for DM.^2^ The reading time required for two-view DBT has been showed to be 38% to 76% longer than for DM, both with narrow-angle^4^^–^^6^ and wide-angle DBT.^7^ If DBT is read together with DM or synthetic mammogram, the increase has been between 73% and 319%.^4^^–^^7^ To the best of our knowledge, no study of reading time for one-view DBT compared with two-view DM is available. However, two studies reported reading times of one sided one-view wide-angle DBT.^8^^,^^9^ These times were similar to the DBT reading times in some of the studies comparing one-sided two-view DBT with DM,^6^^,^^7^ but shorter than some of the studies.^4^^,^^5^ Thus, the reading time of one-view wide-angle DBT might be slightly shorter than two-view DBT, but still longer than two-view DM; however, comparison between studies might be complicated due to different study designs. The longer reading time, together with the limited availability of equipment and higher total cost for DBT, are all barriers for a wider implementation.^10^^,^^11^ Also, the organ dose is often higher with DBT,^12^ though this may depend on equipment and way of implementation.

The development of artificial intelligence (AI) for DM and DBT has shown promising results as a decision support tool for the examining radiologist, but also as a stand-alone reader.^13^^–^^20^ In the future, AI may allow a more efficient workflow and reduce the challenges with longer reading time, but further development and validation is necessary.

Individualized breast cancer screening has been proposed to use resources more efficiently and to increase cancer detection.^21^ While DBT has been shown to detect more cancers, DM might be sufficient for many women. One possibility could be to supplement DM with DBT only in selected groups that would clearly benefit from the addition of DBT. A way of individualizing breast cancer screening is to use a breast cancer risk prediction model, and several different models have been developed, based on different factors including individual health information, family history, genetic testing, and breast density.^22^ Collecting additional information from different sources might be challenging, and an approach focusing on radiographic information could be easier to implement. It has been proposed to risk stratify by breast density alone,^23^ and this approach to selectively add magnetic resonance imaging has been shown to decrease interval cancers.^24^ If only taking breast density into account, other important factors might be missed. A recently presented image-based AI risk model, which also analyzes other characteristics, has shown promising results.^25^ Another model combined different clinical information with results from a system developed for cancer detection.^26^

Recalling healthy women from screening due to false-positive results leads to anxiety, but the level of anxiety depends on the invasiveness of the procedures performed at recall work-up.^27^ There has been concerns that using a risk stratified screening could have a similar effect on those women—of which the majority would still be healthy—who are sorted into the higher risk groups.^28^ However, informing women about a cancer risk estimate has been shown not to cause any major emotional harm, and this might have some similarities with adding DBT in selected cases as this could be perceived as indicating a higher cancer risk.^29^

If women who might benefit from added DBT could be promptly identified based on the characteristics of the corresponding DM image, DBT could be performed directly in conjunction with the DM. Thus, it would not be necessary for the woman to have an additional screening appointment, and there would only be a minor difference in the screening experience between women in high-risk groups compared to the general screening population, which probably could help minimize the increase in anxiety. As both DM and DBT would be present for reading at once, the efficiency of the screen reading would also likely be higher than reading at separate occasions. If DBT would instead be added at an extra appointment after a first radiologist review of the DM and AI results, e.g., in cases with high AI scores but no obvious findings, the number of DBT would likely be slightly lower. However, as this study aimed to setup a workflow that is as streamlined as possible for the radiologists and minimizes unnecessary distress for the women, it was decided to focus on the addition of DBT directly during the screening appointment.

Our group has previously investigated the possibility of using AI to exclude normal cases from human reading,^30^ and also if AI can be used to detect additional cancers on DM that radiologists only detected on DBT.^31^ We have also studied if AI analyzing DBT can be used to make the DBT reading workflow more efficient.^32^ In this study, we investigate whether an AI system designed for breast cancer detection can be used to identify high gain DM cases that would benefit from additional DBT imaging at the same screening occasion. The effect will be quantified in terms of number of additional detected cancers, proportion of women examined with DBT, detection rate, and false positive recalls. Also, the effects on organ dose will be studied.

## 2 Methods

### 2.1 Study Population

**Fig. 1:**
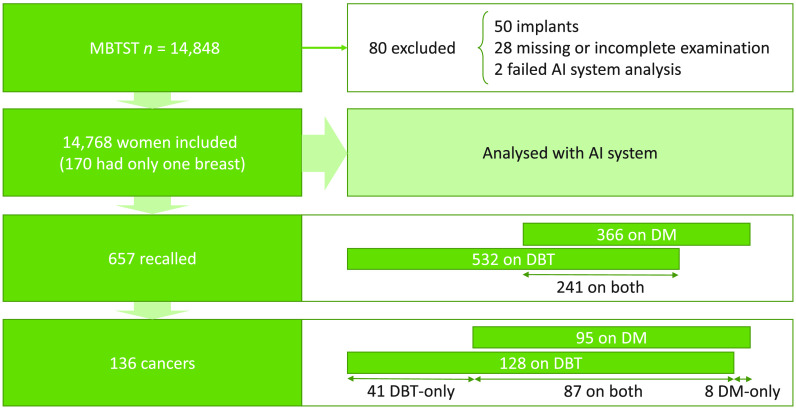
Overview of the study population including exclusions, recalls, and cancers.

We used data from the prospective population-based study Malmö Breast Tomosynthesis Screening Trial (MBTST).^33^ In MBTST, 14,848 women were screened with both two-view DM (CC + MLO) and one-view wide-angle DBT (MLO). All examinations were performed with Siemens Mammomat Inspiration. The examinations were double read in separate reading arms, including separate decisions to recall or not after consensus. A separate step in the DBT reading arm included the DM CC-view, but the effect on the results was very minor compared to DBT only, and thus this aspect has not been included in this study. No computer aided detection system was used in the reading setting. Further, no synthetic mammograms (SM) were used, as this was not available for the mammography system at the time of initiation of the study. A few women had to be excluded from this study, most due to breast implants, which the AI system is not trained to classify, and image data being unavailable for processing. Thus, the present study includes 14,768 women. The study population including exclusions, recalls, and cancers is illustrated in Fig. 1. In total, 136 women were diagnosed with breast cancer, including 128 detected on DBT and 95 detected on DM. Of the cancers, 41 were only detected on DBT and 8 only detected on DM. This study is covered by the study approval for the MBTST by the Local Ethics Committee at Lund University (Official records number: 2009/770).

### 2.2 AI System

In this study, we analyzed the DM examinations with a pre-release version of the deep convolutional neural network-based mammography analysis system Transpara v1.7 (ScreenPoint Medical, Nijmegen, The Netherlands).^13^^,^^14^ This system is designed primarily to be used as a support tool for the radiologist while reading screening examinations, but it has also been tested as a stand-alone reader.^14^ The system provides a decimal risk score for each examination, which is rounded up to an integer 1 to 10 and is calibrated to have a roughly even number of cases (10%) for each score in a screening material, and >85% of cancers in group 10. Although not primarily designed for this purpose, this score was used as a basis for risk stratification in this study with the hypothesis that adding a DBT examination for women with high AI risk score would increase the total cancer detection. For the analyses, the decimal score was used.

### 2.3 Study Design

**Fig. 2:**
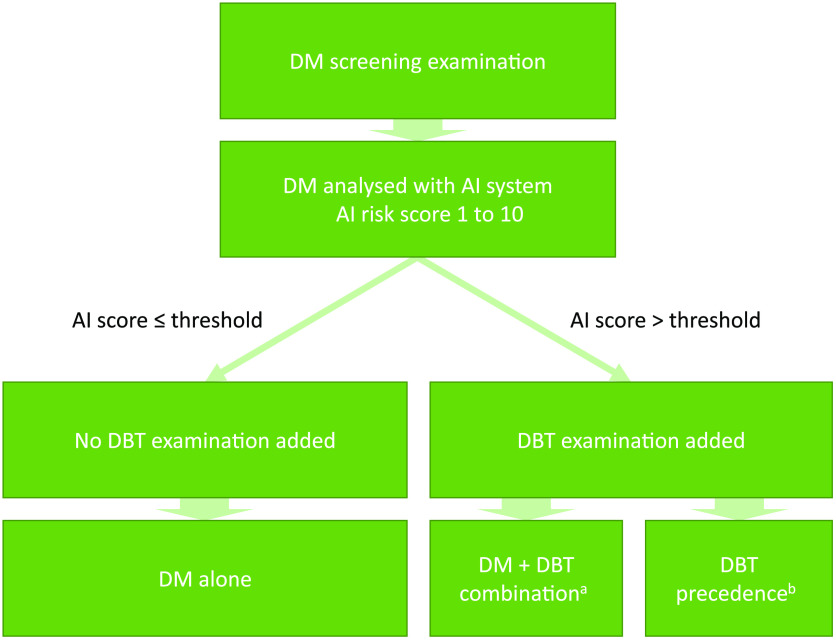
Illustration of study design: (a) combination of DM and DBT with separate double reading and (b) DBT double reading used alone when the AI score is over the threshold.

The design of this study is illustrated in Fig. 2. The DM examinations from all included women were analyzed with the AI system and different thresholds of the AI risk score were tested for use as a discriminator for adding a DBT examination. The original reading data from the MBTST were used in this study, and thus no information about the AI results was available to the radiologists. To make the radiologist reading as efficient as possible, we studied two different workflows for cases where a DBT examination has been added.

1. DM + DBT combination, where the results from the DBT double reading were combined with the results from the DM double reading.
2. DBT precedence, where only the DBT double reading results were used when a DBT examination had been added, which would minimize the increase of the reading workload.

### 2.4 Threshold for Adding DBT and Its Consequences

The number of additionally detected cancers was calculated at different thresholds for adding DBT as a complement to DM. The proportion of women who would be examined with DBT depending on threshold was calculated. The detection rate and the number of false positive recalls were investigated for different thresholds. Two thresholds, 7.5 and 9.0, were selected to study two different proportions of examinations where DBT has to be read, and were evaluated more thoroughly. False positives were defined as women recalled for further examinations due to findings on examinations that would be performed and read in the simulated workflow, without diagnosing a cancer.

### 2.5 Cancer Characteristics for Detected and Missed Cancers

For two different AI score thresholds, we investigated the cancer types of both the extra detected cancers in comparison with only using DM, and the missed cancers in comparison with screening all women with DBT.

### 2.6 Effects on Organ Dose

The organ dose averaged over all the women with added DBT was calculated and compared with the organ dose for DM and DBT alone. The dose calculations were based on the organ dose attribute extracted from the examination metadata. Also, the average organ dose at the population level was calculated for different threshold of adding DBT.

### 2.7 Statistical Analysis

All statistical analyses were performed in MATLAB (The MathWorks, Natick, Massachusetts, United States). Descriptive statistics were used. Further, confidence intervals for the proportions of cancers with different characteristics were calculated at the 95% significance level with the Clopper–Pearson method. For the averaged organ doses, the standard deviation (SD) is reported.

## 3 Results

### 3.1 Threshold for Adding DBT and Following Effects

**Fig. 3:**
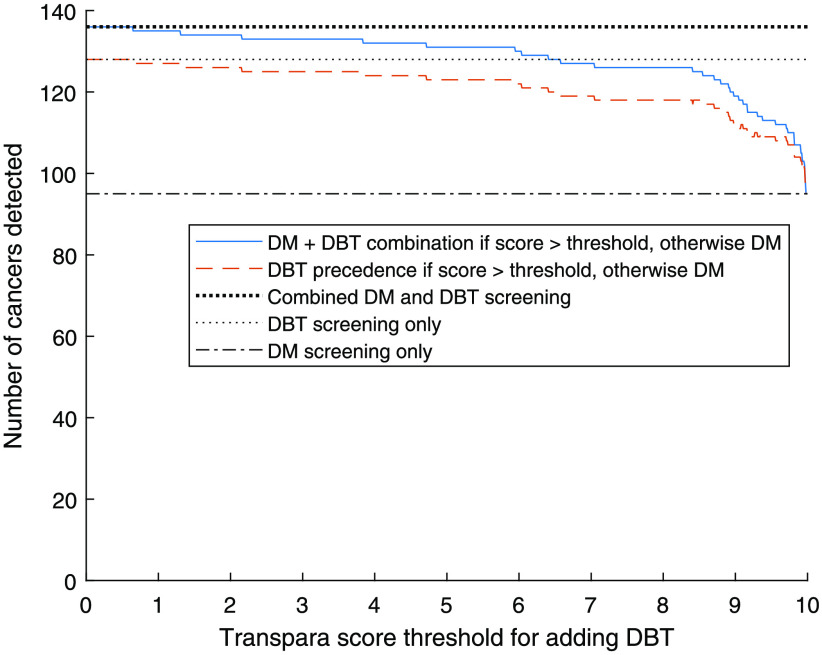
Number of detected cancers depending on AI score threshold for adding DBT to DM.

The number of cancers detected at different AI score thresholds for adding DBT is presented in Fig. 3. With a threshold of 9.0, in total 119 cancers would be detected with the DM + DBT combination workflow. By comparing this with the 95 cancers detected on DM alone, the number of detected cancers is increased by 24 (25%). Of the 41 cancers only detected on DBT, 59% (24/41) would be detected. With the DBT precedence workflow, 7 of the 8 DM-only detected cancers would be missed. The results for thresholds of 9.0 and 7.5, with the DM + DBT combination and DBT precedence approaches respectively, are presented in Table 1.

**Table 1:** Detected and missed cancers for different approaches at different thresholds of adding DBT, compared to DM screening alone.

	Detected cancers			False positives			PPV	DBT exams	DM exams
	Total	Extra	Increase (%)	Total	Extra	Increase (%)		Total	Read
DM only for all cases	95	0	0	271	0	0	0.260	0	14768
DM + DBT combination if AI score > 9.0^a^	119	24	25	331	60	22	0.264	1493	14768
DBT precedence if AI score > 9.0^b^	112	17	18	301	30	11	0.271	1493	13275
DM + DBT combination if AI score > 7.5^a^	126	31	33	374	103	38	0.251	3251	14768
DBT precedence if AI score > 7.5^b^	118	23	24	322	51	19	0.268	3251	11517
DM + DBT^a^ for all cases	136	41	43	521	250	92	0.207	14768	14768
DBT only for all cases	128	33	35	404	133	49	0.241	14768	0^c^

a Data for DM and DBT read together are not available due to limitations in study design.

b DBT performed if AI score > threshold. DM examinations performed for all women, but not read when DBT is available.

c No DM examinations performed.

**Fig. 4:**
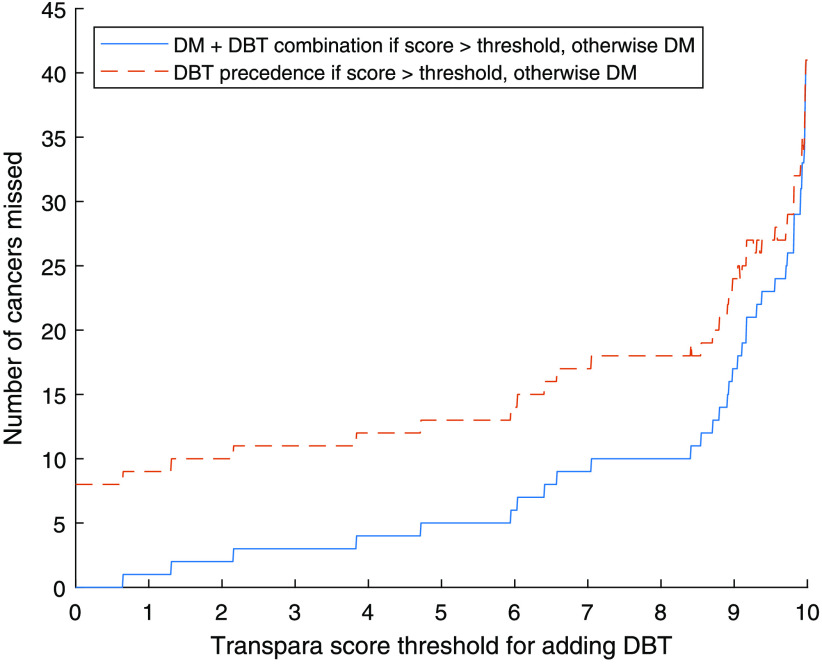
Number of cancers missed in relation to AI score threshold for adding DBT, compared to screening all with both DM and DBT.

The number of cancers missed compared to screening all with both DM and DBT is presented in Fig. 4. If using 9.0 as threshold for adding DBT with DM + DBT combination, 17 cancers (13%) would be missed, and if using 7.5 as threshold 10 cancers (7%) would be missed. Corresponding values with DBT precedence would be 24 missed cancers with 9.0 and 18 missed cancers with 7.5.

**Fig. 5:**
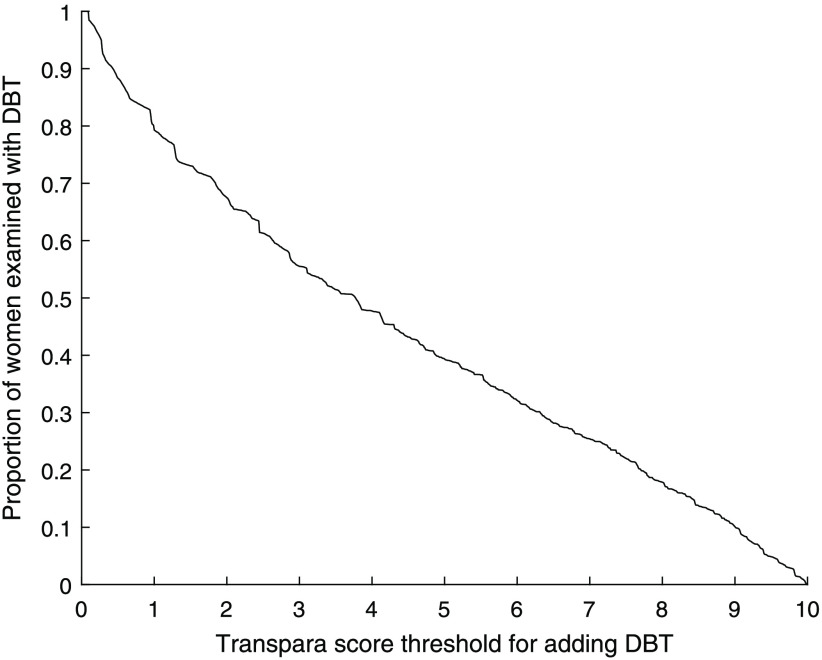
Proportion of women with DBT added depending on AI score threshold.

Figure 5 shows the proportion of the screened women who would be examined with DBT in addition to DM depending on threshold. For a threshold of 9, 1493 (10%) of the screened women would be examined with DBT in addition to DM. If instead a threshold of 7.5 is used, 3251 (22%) women would be examined with DBT.

**Fig. 6:**
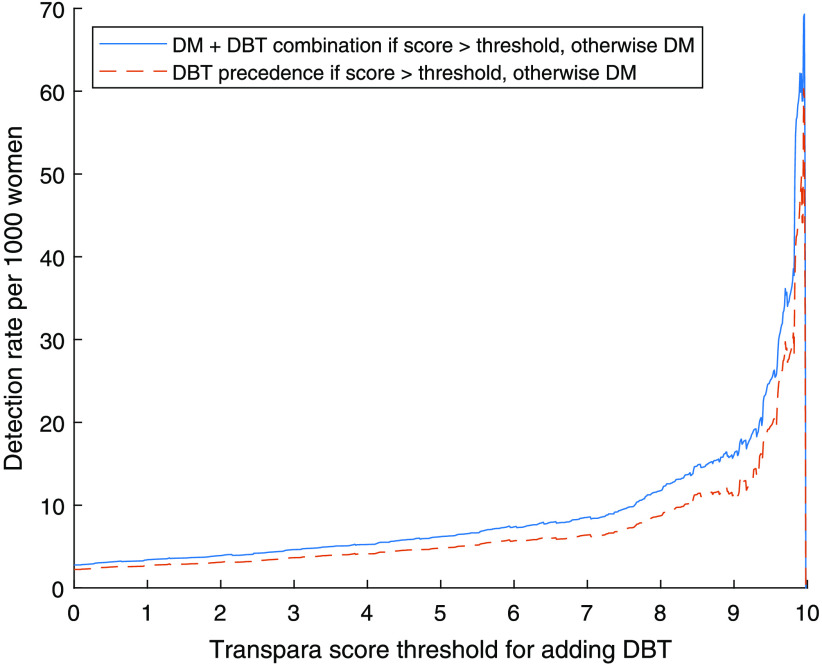
Detection rate for the added DBT examinations in relation to AI score threshold for addition.

The number of detected cancers per 1000 DBT examinations (detection rate) is shown in Fig. 6. With a threshold of 9.0, the number of extra detected cancers per 1000 women with DBT added is 16.1 (24/1493) with DM + DBT combination. For comparison, the total detection rate with a threshold of 9.0 with DM + DBT combination is 8.1 (119/14,768). A threshold of 7.5 would detect 9.54 (31/3251) extra cancers per 1000 women with DBT added. DBT precedence yields slightly lower values i.e., 11.4 (17/1493) extra detected cancers per 1000 women with DBT added with a total detection rate of 7.6 (112/14,768) cancers per 1000 screened women at a threshold of 9.0.

**Fig. 7:**
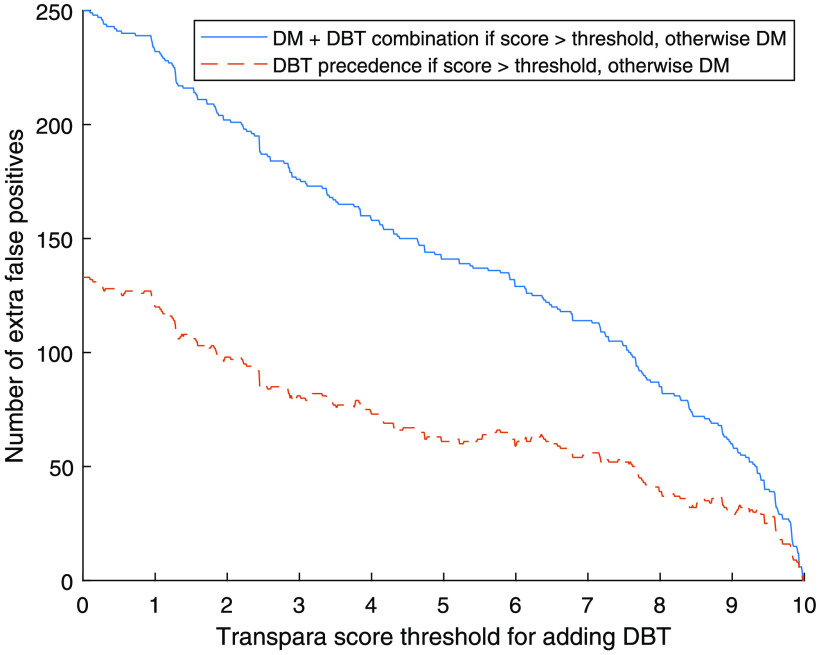
Number of extra false positives depending on threshold for adding DBT.

In Fig. 7 and Table 1, the number of extra false positives compared to screening only with DM, depending on the threshold for adding DBT, is presented. A threshold of 9.0 would add 60 false positives to the 271 false positives for DM only (22% increase) with DM + DBT combination. With DBT precedence, the number of additional false positives would be 30 (11% increase compared to DM). With 7.5 as threshold, the number of extra false positives compared to DM would be 103 (38% increase). This is still less than the 133 (49%) extra false positives with full DBT screening. Table 1 also contains the positive predictive value (PPV) for each of the approaches, which in most cases are higher for the AI approaches compared to full screening with DM screening, DBT, or both.

**Fig. 8:**
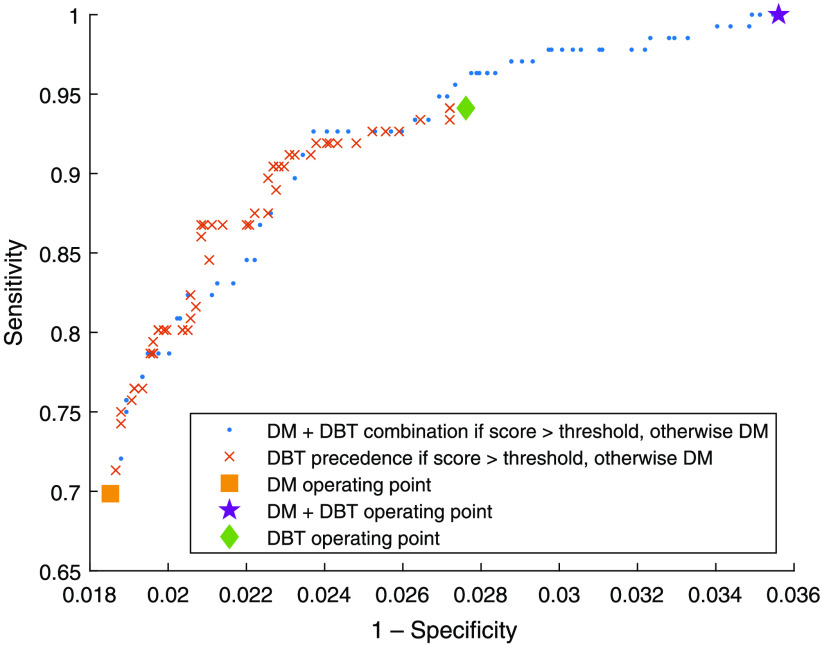
Sensitivity in relation to specificity at different thresholds for adding DBT. For reference, operating points for DM double reading, DBT double reading, and combination of separate DM and DBT double reading, are also shown.

The sensitivity in relation to specificity at different levels of threshold for adding DBT is presented in Fig. 8. The curve is limited by the operating points of radiologist double reading for DM, DBT, and combination of the separate DM and DBT double readings, respectively.

#### 3.2 Cancer Characteristics for Detected and Missed Cancers

Table 2 presents the characteristics of the additional cancers detected if using a threshold of 9.0 or 7.5 with DM + DBT combination workflow, compared to DM screening, and the corresponding missed cancers compared to screening all with both DM and DBT.

**Table 2:** Cancer characteristics for detected and missed cancers depending on chosen strategy.

				DBT added if score > 9.0						DBT added if score > 7.5					
	All cancers	Cancers detected on DM		All detected cancers		Extra detected cancers		Missed cancers		All detected cancers		Extra detected cancer		Missed cancers	
All	136	95	70% [0.61; 0.77]	119	88% [0.81; 0.93]	24	18% [0.12; 0.25]	17	13% [0.07; 0.19]	126	93% [0.87; 0.96]	31	23% [0.16; 0.31]	10	7% [0.04; 0.13]
Invasive	116	79	68% [0.59; 0.76]	102	88% [0.81; 0.93]	23	20% [0.13; 0.28]	14	12% [0.07; 0.19]	107	92% [0.86; 0.96]	28	24% [0.17; 0.33]	9	8% [0.04; 0.14]
*In situ*	20	16	80% [0.56; 0.94]	17	85% [0.62; 0.97]	1	5% [0.00; 0.25]	3	15% [0.03; 0.38]	19	95% [0.75; 1.00]	3	15% [0.03; 0.38]	1	5% [0.00; 0.25]
BIRADS 4th Ed breast density category (per woman)															
1	10	6	60% [0.26; 0.88]	9	90% [0.55; 1.00]	3	30% [0.07; 0.65]	1	10% [0.00; 0.45]	9	90% [0.55; 1.00]	3	30% [0.07; 0.65]	1	10% [0.00; 0.45]
2	43	29	67% [0.51; 0.81]	39	91% [0.78; 0.97]	10	23% [0.12; 0.39]	4	9% [0.03; 0.22]	40	93% [0.81; 0.99]	11	26% [0.14; 0.41]	3	7% [0.01; 0.19]
3	63	49	78% [0.66; 0.87]	56	89% [0.78; 0.95]	7	11% [0.05; 0.22]	7	11% [0.05; 0.22]	59	94% [0.85; 0.98]	10	16% [0.08; 0.27]	4	6% [0.02; 0.15]
4	20	11	55% [0.32; 0.77]	15	75% [0.51; 0.91]	4	20% [0.06; 0.44]	5	25% [0.09; 0.49]	18	90% [0.68; 0.99]	7	35% [0.15; 0.59]	2	10% [0.01; 0.32]
Histological type															
Invasive ductal cancer	74	52	70% [0.59; 0.80]	65	88% [0.78; 0.94]	13	18% [0.10; 0.28]	9	12% [0.06; 0.22]	69	93% [0.85; 0.98]	17	23% [0.14; 0.34]	5	7% [0.02; 0.15]
Invasive lobular cancer	24	14	58% [0.37; 0.78]	20	83% [0.63; 0.95]	6	25% [0.10; 0.47]	4	17% [0.05; 0.37]	21	88% [0.68; 0.97]	7	29% [0.13; 0.51]	3	13% [0.03; 0.32]
Tubular cancer	17	12	71% [0.44; 0.90]	16	94% [0.71; 1.00]	4	24% [0.07; 0.50]	1	6% [0.00; 0.29]	16	94% [0.71; 1.00]	4	24% [0.07; 0.50]	1	6% [0.00; 0.29]
Ductal carcinoma *in situ*	20	16	80% [0.56; 0.94]	17	85% [0.62; 0.97]	1	5% [0.00; 0.25]	3	15% [0.03; 0.38]	19	95% [0.75; 1.00]	3	15% [0.03; 0.38]	1	5% [0.00; 0.25]
Other invasive cancer	1	1	100% [0.03; 1.00]	1	100% [0.03; 1.00]	0	0% [0.00; 0.98]	0	0% [0.00; 0.98]	1	100% [0.03; 1.00]	0	0% [0.00; 0.98]	0	0% [0.00; 0.98]
Histological grade, invasive cancers															
Histological grade 1	45	31	69% [0.53; 0.82]	38	84% [0.71; 0.94]	7	16% [0.06; 0.29]	7	16% [0.06; 0.29]	40	89% [0.76; 0.96]	9	20% [0.10; 0.35]	5	11% [0.04; 0.24]
Histological grade 2	52	34	65% [0.51; 0.78]	46	88% [0.77; 0.96]	12	23% [0.13; 0.37]	6	12% [0.04; 0.23]	49	94% [0.84; 0.99]	15	29% [0.17; 0.43]	3	6% [0.01; 0.16]
Histological grade 3	17	13	76% [0.50; 0.93]	16	94% [0.71; 1.00]	3	18% [0.04; 0.43]	1	6% [0.00; 0.29]	16	94% [0.71; 1.00]	3	18% [0.04; 0.43]	1	6% [0.00; 0.29]
Missing	2	1	50% [0.01; 0.99]	2	100% [0.16; 1.00]	1	50% [0.01; 0.99]	0	0% [0.00; 0.84]	2	100% [0.16; 1.00]	1	50% [0.01; 0.99]	0	0% [0.00; 0.84]
Nuclear grade, *in situ* cancers															
Nuclear grade 1	2	1	50% [0.01; 0.99]	1	50% [0.01; 0.99]	0	0% [0.00; 0.84]	1	50% [0.01; 0.99]	1	50% [0.01; 0.99]	0	0% [0.00; 0.84]	1	50% [0.01; 0.99]
Nuclear grade 2	7	5	71% [0.29; 0.96]	5	71% [0.29; 0.96]	0	0% [0.00; 0.41]	2	29% [0.04; 0.71]	7	100% [0.59; 1.00]	2	29% [0.04; 0.71]	0	0% [0.00; 0.41]
Nuclear grade 3	11	10	91% [0.59; 1.00]	11	100% [0.72; 1.00]	1	9% [0.00; 0.41]	0	0% [0.00; 0.28]	11	100% [0.72; 1.00]	1	9% [0.00; 0.41]	0	0% [0.00; 0.28]
Size, pathology size															
Size ≤10	49	33	67% [0.52; 0.80]	44	90% [0.78; 0.97]	11	22% [0.12; 0.37]	5	10% [0.03; 0.22]	47	96% [0.86; 1.00]	14	29% [0.17; 0.43]	2	4% [0.00; 0.14]
Size 11 to 15	44	29	66% [0.50; 0.80]	37	84% [0.7; 0.93]	8	18% [0.08; 0.33]	7	16% [0.07; 0.30]	39	89% [0.75; 0.96]	10	23% [0.11; 0.38]	5	11% [0.04; 0.25]
16 to 19	14	10	71% [0.42; 0.92]	12	86% [0.57; 0.98]	2	14% [0.02; 0.43]	2	14% [0.02; 0.43]	13	93% [0.66; 1.00]	3	21% [0.05; 0.51]	1	7% [0.00; 0.34]
≥20	27	22	81% [0.62; 0.94]	24	89% [0.71; 0.98]	2	7% [0.01; 0.24]	3	11% [0.02; 0.29]	25	93% [0.76; 0.99]	3	11% [0.02; 0.29]	2	7% [0.01; 0.24]
Missing	2	1	50% [0.01; 0.99]	2	100% [0.16; 1.00]	1	50% [0.01; 0.99]	0	0% [0.00; 0.84]	2	100% [0.16; 1.00]	1	50% [0.01; 0.99]	0	0% [0.00; 0.84]
Axillary lymph node status															
Lymph node positive	29	21	72% [0.53; 0.87]	27	93% [0.77; 0.99]	6	21% [0.08; 0.40]	2	7% [0.01; 0.23]	28	97% [0.82; 1.00]	7	24% [0.10; 0.44]	1	3% [0.00; 0.18]
Lymph node negative	94	63	67% [0.57; 0.76]	80	85% [0.76; 0.92]	17	18% [0.11; 0.27]	14	15% [0.08; 0.24]	86	91% [0.84; 0.96]	23	24% [0.16; 0.34]	8	9% [0.04; 0.16]
Missing	13	11	85% [0.55; 0.98]	12	92% [0.64; 1.00]	1	8% [0.00; 0.36]	1	8% [0.00; 0.36]	12	92% [0.64; 1.00]	1	8% [0.00; 0.36]	1	8% [0.00; 0.36]
Radiographic appearance															
Spiculated mass	91	62	68% [0.58; 0.78]	78	86% [0.77; 0.92]	16	18% [0.10; 0.27]	13	14% [0.08; 0.23]	83	91% [0.83; 0.96]	21	23% [0.15; 0.33]	8	9% [0.04; 0.17]
Circumscribed mass	18	11	61% [0.36; 0.83]	15	83% [0.59; 0.96]	4	22% [0.06; 0.48]	3	17% [0.04; 0.41]	17	94% [0.73; 1.00]	6	33% [0.13; 0.59]	1	6% [0.00; 0.27]
Microcalcifications	22	20	91% [0.71; 0.99]	22	100% [0.85; 1.00]	2	9% [0.01; 0.29]	0	0% [0.00; 0.15]	22	100% [0.85; 1.00]	2	9% [0.01; 0.29]	0	0% [0.00; 0.15]
Distortion	4	1	25% [0.01; 0.81]	3	75% [0.19; 0.99]	2	50% [0.07; 0.93]	1	25% [0.01; 0.81]	3	75% [0.19; 0.99]	2	50% [0.07; 0.93]	1	25% [0.01; 0.81]
Lymph node	1	1	100% [0.03; 1.00]	1	100% [0.03; 1.00]	0	0% [0.00; 0.98]	0	0% [0.00; 0.98]	1	100% [0.03; 1.00]	0	0% [0.00; 0.98]	0	0% [0.00; 0.98]

Results for DM + DBT combination workflow, i.e. DM and DBT double readings are combined when the AI score is over the threshold, otherwise only the DM reading is used. Values in brackets are 95% confidence intervals calculated with the Clopper–Pearson method.

**Fig. 9:**
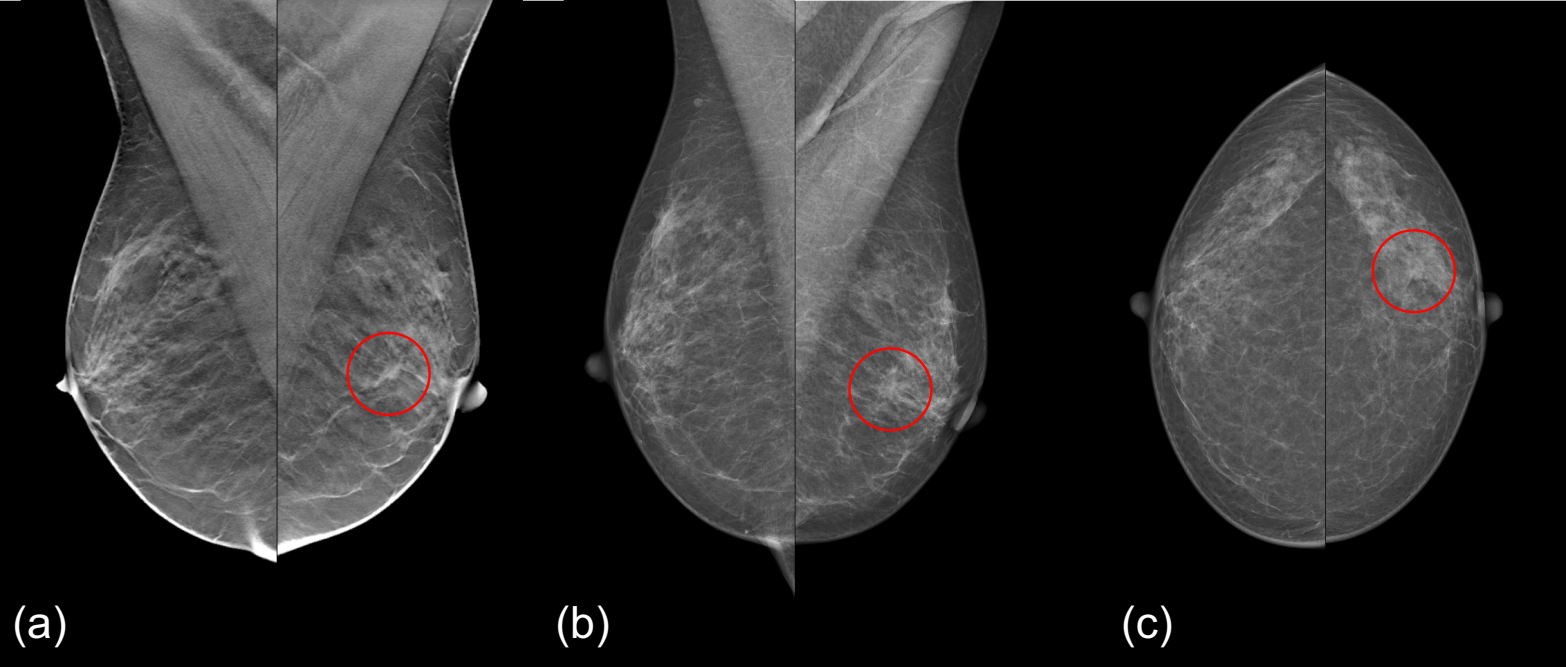
Cancer (red circle) missed with DM screening, but detected with selective addition of DBT as the examination got an AI score of 9.91. The cancer was a 7 mm invasive ductal carcinoma. (a) MLO DBT, (b) MLO DM, and (c) CC DM.

**Fig. 10:**
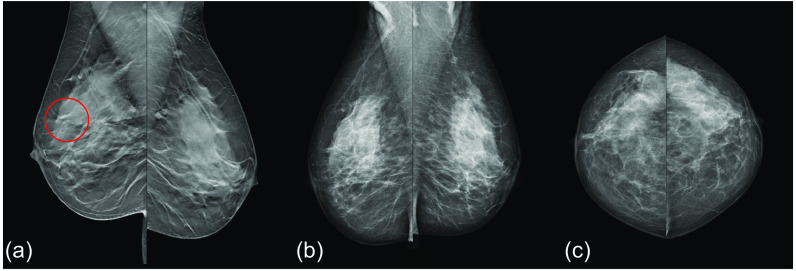
Cancer (red circle) detected with double reading DBT screening, but not detected at double reading DM screening nor detected with selective addition of DBT. The examination got an AI score of 4.72 and would thus be missed regardless of if a threshold of 7.5 or 9.0 is used. The cancer was a 25 mm invasive lobular carcinoma. (a) MLO DBT, (b) MLO DM, and (c) CC DM.

An example of a cancer that would be missed with DM screening, but detected when adding DBT when the AI score is over 9.0, is shown in Fig. 9. Figure 10 presents an example of a cancer that was detected with DBT screening, but missed with DM screening and when only adding DBT if AI score is over 7.5.

#### 3.3 Effects on Organ Dose

If only using DM screening (one CC and one MLO view per breast) the organ dose averaged over all examinations was 2.69 mGy (SD: 0.778) and if only DBT (one single MLO view per breast) was used the organ dose average was 2.24 mGy (SD: 0.672). The women examined with both DM and DBT would have an organ dose of 4.91 mGy (SD: 1.347) on average. The organ dose average at the population level would be 2.89 mGy (SD: 1.072) if DBT is added when the AI score is >9.0, and 3.14 mGy (SD: 1.316) if DBT is added when the AI score is >7.5.

## 4 Discussion

We have investigated the possibility of using the score from an AI cancer detection system applied on DM for risk stratification to select high gain cases for further examination with DBT in a retrospective setting. We found that using a threshold of 9.0, 12% of the women would have DBT added, and with DM + DBT combination 25% more cancers would be detected, at a cost of 22% increase in false positives. The PPV is slightly increased compared to DM screening, instead of a decrement as seen with full DBT screening. If instead the DBT precedence approach is used, i.e., DBT is used alone when the score is over the threshold, 18% more cancers would be detected, compared to DM only, at a cost of just 11% increase in false positives. With DBT-only screening for all women, the corresponding increase in cancer detection would be 35% with 49% more false positives. Out of the 41 cancers only detected with DBT screening, 59% would be detected by adding DBT for women with DM scored over a threshold of 9.0.

### 4.1 Threshold for Adding DBT and Following Effects

The level of the threshold has a critical impact on the number of detected cancers, and a lower threshold would lead to more detected cancers, with of course a maximum at the level of adding DBT to all women. While the effect on the number of detected cancers is highest among the higher scores, relatively few cancers would be gained by lowering the threshold below 7.5 (Fig. 3). The proportion of women examined with DBT (Fig. 5) has an almost linear relationship to the threshold, as expected since the system is calibrated by the manufacturer to have about 10% with each score in a screening material (though the exact proportions can conceivably vary with different populations). Also, the number of false positives (Fig. 7) for scores lower than about 7.5 has an almost linear relationship to the threshold. Both the number of DBT and number of false positives are associated with high health care costs and demand more health care resources, but also have costs in health and well-being for unnecessarily recalled women. Several aspects should be taken into account when deciding the level of the threshold, and this could also be different depending on available resources. We have decided to focus on the thresholds 9.0, which with relatively few resources would detect many of the potential cancers, and 7.5, which would find more cancers but use more resources.

The detection rate for the selectively added DBT, of 16.1 per 1000 women screened when combining DM and DBT when indicated by a score over 9.0 (Fig. 6), can be compared with the detection rates for the original study of 6.5 for DM and 8.7 for DBT, respectively.^22^ The large difference stresses the need of focusing limited resources on the high gain groups to maximize the results in order of cancer detection. The total detection rate of 8.1/1000 women screened (DM + DBT combination with threshold 9.0) is less than the detection rate when screening all women with DBT (8.7/1000) and from the perspective of cancer detection alone a full DBT screening program would be a better solution, with the possible caveats of an increased false positive rate and increased overdiagnosis.

### 4.2 DM + DBT Combination or DBT Precedence

DM + DBT combination, combining results from DM and DBT double reading, will obviously detect the most cancers, but also other aspects must be taken into account. In Fig. 8, the receiver operating characteristic-curve when using the DBT precedence approach has a slightly steeper incline than the curve for combining DM and DBT results, indicating that a higher sensitivity in relation to specificity can be achieved using DBT alone when the AI score is over the threshold. This is consistent with the lower number of false positives (Fig. 7). This approach could also lead to a slight reduction in the reading time, since the image material would be smaller. However, for comparison with any prior DM examinations, in a clinical situation it would probably be valuable to also use the DM.

Due to the study design of the MBTST with two arms double reading DM and DBT, respectively, with separate decisions to recall, it is not possible to *a posteriori* determine how the result would be if both the DM and DBT examinations were read at the same time. Probably at least some recalls at DM could be avoided, if areas looking suspicious due to overlaying of normal structures could be studied also on a corresponding DBT examination. Also, some recalls at DBT could be avoided if comparison with prior DM examinations could be made easier with corresponding DM examinations, with the caveat of missing cancers only visible on DBT if too much confidence is assigned to the DM examination. The two separate double readings also mean that up to four different radiologists have read the examinations from each woman, either on DM or DBT, which is not realistic in a real screening workflow. It is reasonable that a prospective study with DM and DBT double read at the same time would yield results somewhere in between the DM + DBT combination and DBT precedence workflows investigated in this study.

### 4.3 Cancer Characteristics for Detected and Missed Cancers

The small number of cancers in each characteristics group makes it hard to draw any conclusions about differences, but there are no obvious differences between all the detected cancers, the extra detected cancers and missed cancers, respectively. Most of the extra detected cancers, as well as the missed cancers, were invasive cancer types.

### 4.4 Effects on Organ Dose

The addition of DBT would lead to a higher organ dose to the selected women, but the difference would be small on the population level regardless of if an AI score threshold of 9.0 or 7.5 is used. Since the detection rate for the extra DBT is substantially higher than for the screening program as a whole, the examination is therefore at least as justifiable. One way of keeping the organ dose as low as possible would be to use only DBT without DM for the upcoming screening rounds for the women who once have been examined with DBT.

Compared to using DBT in conjunction with DM in the whole screening population, a selective addition of DBT for women with the highest gain would lead to a lower organ dose at a population level. A complete move away from two-view DM to one-view DBT has also been proposed,^22^ which would decrease the organ dose compared to DM screening if used for all women.

### 4.5 Effects on Reading Time

Since reading time was not measured in the MBTST, it is not possible to calculate the actual difference in reading time depending on AI score threshold. The generalizability of results from other studies is uncertain since all studies comparing reading time between DM and DBT used two-view DBT, some included only one breast, and some had cancer-enriched materials. As a relatively conservative approximation, an increase in reading time of about 75% is reasonable when DBT is examined instead of DM, and about 125% when both DM and DBT is read together. With an AI score threshold for adding DBT of 9.0 the total screening reading time would increase by 11% with DM + DBT combination, or 6% with DBT precedence. With a threshold of 7.5, corresponding increase in reading time would be 27% and 16%, respectively. An increase of the reading time of, e.g., 6% is relatively small and could probably be handled with only slightly increased resources.

### 4.6 Clinical Implications

The extra detected cancers represent part of the additional cancers detected with DBT screening, which has been shown to be followed by a reduced interval cancer rate. Thus, screening detection of the extra cancers likely has an important clinical value.^34^ Most of the cancers missed with the proposed approach were invasive and it is possible that some of these would appear as interval cancers.

Using DBT only in selected high gain cases could be an alternative to using DBT in the whole screening population and could be more feasible and cost-effective. There may be several logistical challenges at screening centers, since there would be a larger variation in the examination time and it would also be necessary to have DBT capability at all screening centers. Screening with only one-view DBT might have a more uniform examination time and in some aspects cause less logistical challenges and could detect even more cancers than DM with selective addition of DBT but with less radiation. AI systems for analyzing DBT examinations still need further developments and clinical validation, but might open possibilities for reducing the reading time for DBT screening in the future.^17^^,^^35^ If the issues with longer reading time can be handled, a complete DBT screening is probably preferable, if possible with respect to equipment and resources.

While the reading time of screening examinations is important for the workload due to the large number of screening cases, recalled cases usually require far more time, and thus even a small increase in recalls would have a large impact on the total workload. Thus, it is crucial to minimize the number of false positive recalls. Both the DM + DBT combination and the DBT precedence workflows have lower false positive recall rates than full DBT screening, but still this is higher than DM screening and could be an obstacle for clinical implementation of at least some of the studied workflows.

Even a selective addition of DBT in high gain cases would lead to a slight increase in number of false positives, and a few more women would be subject to the anxiety and other risks related to a false positive recall. If the number can be kept low, the gain in cancer detection could probably outweigh this on a population level. Also, the women automatically selected for DBT might feel discomfort and anxiety, due to the knowledge of having a higher breast cancer risk. However, according to results from a prior study, this effect is probably limited.^16^

An alternative implementation could be to use DBT and SM, which have been shown to be equal to DM.^36^^–^^38^ The AI system could then be used to select whether to read the DBT, requiring longer reading time, or if it is sufficient to read only the SM.

### 4.7 Limitations

This study only includes a single center with DM and DBT units from a single vendor, which limits the generalizability. The study is retrospective where the AI system was applied as a stand-alone reader after the trial, and it is therefore not possible to assess if the reading radiologists in a true screening setting would have a different recall behavior if they knew that the AI system has classified the cases with a DBT as high-risk cases. Since the DM and DBT examinations were only read separately, it is not possible to study how reading both the DM and DBT at the same time would affect the results. This could potentially impact both the cancer detection rate and false-positive recall rate. It is necessary to investigate the effects on clinical work in a prospective screening setting, both with respect to outcomes when reading DM and DBT at the same time, and how the use of AI would affect the behavior of radiologists.

The present study used one-view DBT, and it is possible that two views could give better results. To the best of our knowledge, one-view DBT screening has not been used outside of the research setting, and thus two-view DBT might be more likely in a potential clinical implementation of DBT addition for AI selected cases. Some cancers were missed with DBT but detected with DM; however, this is not necessarily due to the absence of a CC-view, but can be related to a number of different factors, including better comparison with previous DM studies in the DM reading arm and inter-reader variations. In this study, combined DM and DBT screening results without respect to interval cancers and cancers detected at next screening round, is used as ground truth. To get a better ground truth, also interval cancers, cancers detected at next screening round and long-time breast cancer mortality should be taken into account. However, this study focused on how DM with selective addition of DBT would perform compared to examining everyone with either DM or DBT specifically in the screening situation, and thus the interval cancers are not in the scope of this study.

Further studies in different populations and using different equipment are necessary to assert generalizability. The proposed selective addition of DBT in high gain cases should be compared prospectively over several screening rounds with DM only, and complete DBT screening, respectively. It should also be investigated if improved results could be achieved by also taking breast density into account or using a system specifically developed for risk stratification.

## 5 Conclusions

If possible with respect to resources, one-view DBT screening could detect more cancers at a lower organ dose than current DM screening. However, breast cancer screening with DM complemented with the addition of DBT in AI selected cases could detect 25% more cancers than DM alone at the cost of 22% extra false positives. Compared to a complete screening with DM and DBT, 59% of the cancers detected only on DBT could be detected by examining only 10% of the women with DBT, with a 36% reduction of false positives. This would be substantially more resource efficient than a complete DBT screening, with a superior PPV. Prospective studies in a clinical setting are necessary.
